# State of malaria diagnostic testing at clinical laboratories in the United States, 2010: a nationwide survey

**DOI:** 10.1186/1475-2875-10-340

**Published:** 2011-11-10

**Authors:** Francisca A Abanyie, Paul M Arguin, Julie Gutman

**Affiliations:** 1 Division of Pediatric Infectious Diseases, Emory University School of Medicine and Children's Healthcare of Atlanta at Egleston, 2015 Uppergate Drive NE, Atlanta, GA 30322, USA; 2 Malaria Branch, Centers for Disease Control and Prevention (CDC), Atlanta, Georgia, USA

**Keywords:** malaria, diagnostic testing, rapid diagnostic tests, United States

## Abstract

**Background:**

The diagnosis of malaria can be difficult in non-endemic areas, such as the United States, and delays in diagnosis and errors in treatment occur too often.

**Methods:**

A nationwide survey of laboratories in the United States and its nine dependent territories was conducted in 2010 to determine factors that may contribute to shortcomings in the diagnosis of malaria. This survey explored the availability of malaria diagnostic tests, techniques used, and reporting practices.

**Results:**

The survey was completed by 201 participants. Ninety percent reported that their laboratories had at least one type of malaria diagnostic test available on-site. Nearly all of the respondents' laboratories performed thick and thin smears on-site; approximately 50% had access to molecular testing; and only 17% had access to rapid diagnostic tests on-site. Seventy-three percent reported fewer than five confirmed cases of malaria in their laboratory during the 12-month period preceding the survey. Twenty-eight percent stated that results of species identification took more than 24 hours to report. Only five of 149 respondents that performed testing 24 hours a day, 7 days a week complied with all of the Clinical and Laboratory Standards Institute (CLSI) guidelines for analysis and reporting of results.

**Conclusion:**

Although malaria diagnostic testing services were available to a majority of U.S. laboratories surveyed, very few were in complete compliance with all of the CLSI guidelines for analysis and reporting of results, and most respondents reported very few cases of malaria annually. Laboratories' difficulty in adhering to the rigorous CLSI guidelines and their personnel's lack of practice and proficiency may account for delays and errors in diagnosis. It is recommended that laboratories that infrequently process samples for malaria seek opportunities for practice and proficiency training annually and take advantage of available resources to assist in species identification.

## Background

Malaria continues to be endemic in more than 100 countries worldwide, where it remains a leading cause of morbidity and mortality [1]. Millions of U.S. travellers venture to endemic countries annually [2]. An average of 1,500 cases and five deaths due to malaria occur annually in the U.S. These numbers include U.S. travellers to endemic countries as well as foreign visitors diagnosed and treated in the U.S. [3-7]. A total of 19 malaria-related deaths were reported in the U.S. between 2004 and 2008; diagnostic delay was a contributing factor in at least six [3-7]. Prompt and accurate diagnosis and timely treatment are crucial in reducing malaria-associated morbidity and mortality [8].

**Figure 1:**
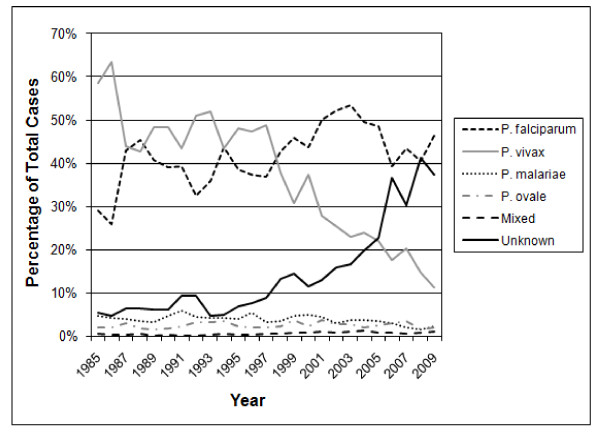
**Malaria cases by species, 1985-2009**. The percentage of cases reported as unknown species has increased considerably in recent years.

In a case series of imported malaria in the late 1990s in Canada, up to 92% of patients who presented to physicians without expertise in tropical medicine experienced delays in diagnosis due to physician failure to consider malaria as a diagnosis on initial presentation, laboratory error in recognition and species identification, or administration of incorrect therapy to treat the parasite(s) identified [9]. In this study, the majority of community laboratories did not perform malaria smears on an urgent basis, nor did they routinely report species identification or percent parasitaemia [9]. Overall, these laboratories had significant reporting delays, misdiagnoses and incorrect species identification [9]. Additionally, proficiency testing in the U.S. in the last decade has revealed especially poor identification of non-falciparum species of malaria [10]. A decline in number of cases reported that include species identification has also been seen in the malaria surveillance data reported to the Centers for Disease Control and Prevention (CDC) (Figure 1) [3-7].

The Clinical and Laboratory Standards Institute's (CLSI) recommendations for laboratories performing malaria diagnostic testing include [11]:

1. Make diagnostic testing available 24 hours a day, seven days a week.

2. Prepare at least three thick and thin smears.

3. Use Giemsa stain for definitive diagnosis. It allows proper visualization of stippling (Schűffner's dots) in *Plasmodium vivax *and *Plasmodium ovale *and can play a crucial role in identification of these species. Although parasites are visible by Wright's and Wright-Giemsa stains, these stains do not allow visualization of stippling.

4. Examine at least 300 fields using the 100× oil immersion objective.

5. Report microscopy results immediately to the requesting physician or ward.

6. Examine at least 10 fields on the thin film to determine percent parasitaemia; many more fields should be examined for patients from the U.S. who typically have lower parasitaemia (CDC recommends counting parasitized red blood cells (RBCs) among 500-2,000 RBCs on the thin smear [12]).

A preliminary report of positive or negative should be available within four hours; percent parasitaemia should be reported within six hours and species identification within 24 hours.

The focus of this survey was to identify laboratory practices that could contribute to delayed or incorrect diagnoses. The goal was to determine the proportion of laboratories in the United States that perform diagnostic malaria testing using procedures that might contribute to diagnostic delays.

## Methods

A convenience sample of laboratory-related personnel was obtained through a nationwide survey of U.S. laboratories to determine the practices for malaria diagnostic testing, including availability of diagnostic tests, time required for reporting, and test methodologies used (see Additional file 1 for survey questions). An introductory e-mail and two reminder e-mails with an embedded link to a web-based survey were sent to two listservs maintained by the American Society for Microbiology. The listservs include doctoral level microbiology laboratory directors and other members of the microbiology laboratory including pathologists, haematologists, clinical laboratory scientists, and medical technologists worldwide; the survey was limited to individuals residing in the U.S. and its nine dependent territories.

The survey was validated by several members of the laboratory staff at varying education levels including bachelors, masters and doctorate level personnel. Survey results were analysed using SurveyMonkey™ and Excel (Microsoft Office 2007, Seattle WA). This study was submitted to the Institutional Review Board of Emory University and deemed exempt from review. Informed consent was implied by response.

## Results

### Study participants

**Figure 2:**
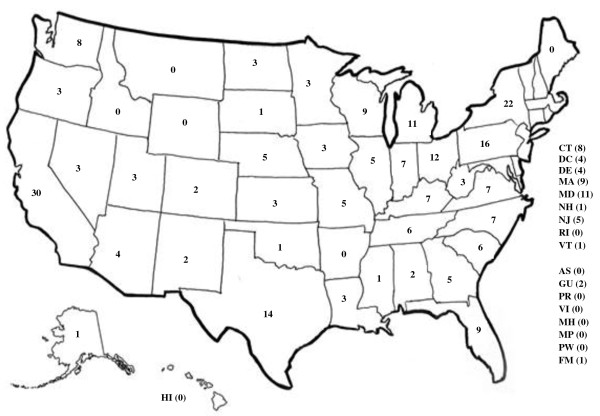
**Number of respondents from each U.S. state or dependent territory**.

A total of 278 participants initiated the survey and 201 (72.3%) completed the survey. Respondents were from laboratories located in 46 of the 59 (78%) states and territories, including 30 respondents in California, 22 in New York, 14 in Texas, and 12 in Ohio (Figure 2). The majority of respondents were microbiologists or microbiology laboratory directors or supervisors; the remainder were haematologists, pathologists, clinical laboratory scientists and medical technologists. Nearly half of respondents described their facility as a community hospital-affiliated laboratory, and fewer than one-third were affiliated with a university hospital (Table 1).

**Table 1:** Classification of laboratories

Laboratory Classification	No. (%)^a^	Percentage of laboratory type with on-site testing available (%)
**Community hospital**	130 (46.8)	87
**University hospital**	83 (29.8)	100
**Commercial referral laboratory**	21 (7.6)	90
**Public health**	15 (5.4)	67
**VA hospital**	9 (3.2)	89
**Primary care center**	4 (1.4)	75
**Other**	16 (5.8)	88
**TOTAL**	278	

^a ^Includes all respondents that started survey, regardless of completion status.

### Availability of diagnostic testing

Ninety percent of all respondents worked in a laboratory with at least one type of malaria diagnostic test available on-site. This included all respondents from university hospital laboratories, 87% from community hospital-affiliated laboratories, 90% from commercial referral laboratories, and 88% from other laboratories (Table 1). The remaining 10% sent all specimens to an outside laboratory for analysis.

### Laboratories with on-site malaria diagnostics

Of the respondents who reported that their laboratory performed diagnostic testing for malaria, 149 (85%) reported that their laboratory provided diagnostic testing either in-house or via on-call personnel, 24 hours a day, seven days a week. This number included 96% of respondents from university hospital laboratories, 83% from commercial referral laboratories, 82% from community hospitals, and 100% from other laboratories. The remaining 15% worked in laboratories with personnel who could perform diagnostic testing only during the 8- to 12-hour work day.

**Figure 3:**
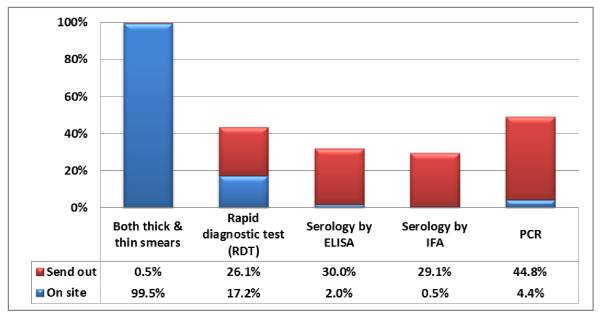
**Availability of Malaria Diagnostic Testing at Laboratories with On-Site Testing**. This graph shows the percentage of tests performed on-site and as send-outs at laboratories with at least one malaria diagnostic test available on-site (N = 203). (ELISA- Enzyme-linked immunosorbent assay, IFA- indirect fluorescent antibody, PCR- polymerase chain reaction).

Sixty-nine respondents (32%) noted that their laboratory received <10 specimens for malaria diagnostic testing within the 12 months preceding the survey. Twenty-seven (13%) received a large volume, with >100 specimens sent for analysis; however, most of these specimens were negative. Only 10 (~5%) had >15 confirmed cases, while 158 (73%) respondents noted that their laboratory had five or fewer confirmed cases of malaria during the 12 months preceding the survey. The type of diagnostic testing available on-site is shown in Figure 3. Nearly all laboratories with on-site testing performed both thick and thin smear analysis by light microscopy. Only 35 respondents (17%) reported using rapid diagnostic testing (RDT); 20 of these were from university hospital laboratories, seven from community hospitals, two from commercial referral laboratories, one from a public health laboratory, and five from other laboratories. PCR was available to about half the respondents, with nine respondents reporting direct access in their laboratory (two university-affiliated laboratories, two commercial referral laboratories, three public health laboratories, and two other laboratories) and an additional 91 respondents with access to PCR as a send-out test.

#### Technique

Of the 202 respondents in laboratories where light microscopy was performed on-site, nearly half (47%) used Giemsa stain in at least one step of the analysis of thick and thin smears; several others used more than one of three staining techniques: Giemsa, Wright-Giemsa, and Wright stain. Two respondents reported using acridine orange stain; in one laboratory, this was the only stain used, and in the other, positive staining with acridine orange was followed by Wright-Giemsa staining.

Eighty-three respondents (46%) reported that their laboratory reviewed ≥three slides from a single patient's sample before a specimen was considered negative; this was true for 44% of university and 53% of community hospital respondents. A minority, 16 (~9%), reported that their laboratory reviewed only one slide to determine a negative test. The majority of respondents, 112 (63%), reported that their laboratory reviewed ≥300 high power fields (HPF) prior to determining that a slide was negative. This was the case in 75% of respondents from commercial referral laboratories, 68% from university hospitals, and 67% from other laboratories; 12 (~7%) reviewed <100 HPF to come to this conclusion.

The majority of respondents, 91 (58%), reported that 500-2,000 RBCs were counted to determine percent parasitaemia. This included 67% of respondents from public health laboratories, 60% from university hospital laboratories, and 71% from other laboratories. Fourteen percent of respondents reported their laboratories counting >2,000 cells, while 14% reported counting <500 cells.

#### Reporting

The majority of respondents, 115 (64%), noted that a preliminary result was reported within 4 hours of receiving the specimen. This was the case for 74% of respondents from university hospitals, 71% from public health laboratories, and 62% from community hospitals. Approximately seven in 10 respondents (126) reported that at least two personnel (in most laboratories, two technologists) reviewed a slide prior to reporting a negative result; the remaining respondents reported that only a single technologist reviewed the slide. In contrast, most respondents, 127 (71%), reported that positive results were confirmed by a pathologist or laboratory director/supervisor. A little over half of the respondents, 93 (52%), noted that their laboratory reported percent parasitaemia within six hours. A majority of respondents from university (59%) and public health (71%) facilities stated that percent parasitaemia was reported within this time period.

A species-specific diagnosis was possible in-house for 144 (89%) respondents. For 133 (70%) respondents, a species-specific diagnosis was made within 24 hours; half of these were made within 6 hours after receipt of specimen. Species identification was available within 6 hours according to 67% of respondents from public health laboratories, 40% from university hospitals, 29% from community hospitals, and 57% from other laboratories. Twenty-eight percent reported that a species diagnosis took >24 hours.

In 102 (63%) respondents' laboratories, species identification was performed in-house by either a microbiology director/supervisor or a pathologist. Approximately 40% noted that species identification was performed in-house and subsequently confirmed at an outside laboratory, such as the state or local health department or CDC. Eleven percent reported that species identification was done only at an outside laboratory.

#### Compliance summary

**Figure 4:**
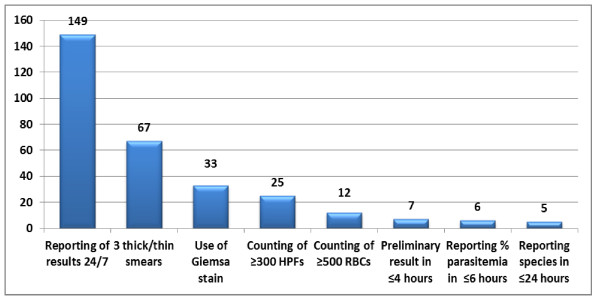
**Sequential Compliance with CLSI Guidelines**.

Of the 149 respondents who reported that their laboratory provided diagnostic testing either in-house or via on-call personnel, 24 hours a day, seven days a week, using the strict definition of immediate preliminary reporting within four hours, percent parasitaemia within six hours and species identification within 24 hours only five were in complete compliance with all six CLSI guidelines (Figure 4). This included three respondents' laboratories affiliated with a university hospital, one from a community hospital, and one from a laboratory categorized as other. If more liberal definitions were used to define immediate reporting (ie: preliminary report within12 hours, percent parasitaemia within 12 hours, and species identification within 36 hours) nine respondents' laboratories were in complete compliance (four from university hospitals, three from community hospitals, one from a commercial referral laboratory, and one from a laboratory categorized as other).

### Laboratories providing only off-site testing

Twenty-eight (10%) respondents reported that their laboratory provided only send-out diagnostic testing for malaria. Seventeen (60%) were affiliated with community hospitals, five (18%) with public health laboratories, and two each with commercial referral and other laboratories. Of the 27 respondents whose laboratories offered only send-out testing for malaria who completed the survey, 85% reported receiving <10 specimens for malaria diagnostic testing within the 12 months preceding the survey. Only one respondent stated his laboratory received >100 specimens. Twenty-five (93%) reported ≤five confirmed cases of malaria; half of these respondents had seen no positive cases of malaria in their laboratory in the past year.

**Figure 5:**
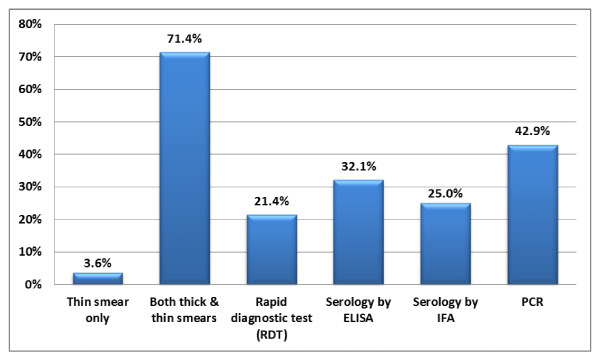
**Availability of Malaria Diagnostic Testing at Laboratories without On-Site Testing**. This graph shows the percentage of respondents from laboratories where all malaria testing is performed off-site reporting access to various tests (N = 28). (ELISA- Enzyme-linked immunosorbent assay, IFA- indirect fluorescent antibody, PCR- polymerase chain reaction).

The diagnostic tests available to the laboratories where only off-site testing is performed are shown in Figure 5. The majority reported that their laboratory had access to thick and thin smears by light microscopy (71%), and some reported access to PCR (43%). Many of these respondents (41%) reported that their laboratories received a report of the level of parasitaemia and speciation within 24 - 48 hours; three respondents reported that results were not available until >7 days after the specimen had been sent out.

## Discussion

It is undeniable that there are significant challenges in diagnosing malaria, especially in areas where it is not endemic. Laboratory challenges in the diagnosis of malaria include patients' low parasite density, altered parasite morphology due to the patients' use of chemoprophylaxis, empiric therapy, mishandling of specimens, and inexperience in identifying malaria parasites due to the small number of cases seen [8]. The combination of these factors potentially reduces the sensitivity of microscopy significantly, making diagnosis very difficult [8].

A recent retrospective review by Edson *et al *analysing the proficiency of U.S. laboratories to identify malaria parasites reported a failure rate of 11.2% for identification of *Plasmodium falciparum *[10]. Although this means that more than one in 10 specimens were not correctly identified, it is encouraging compared with Canadian and British laboratory failure rates of 27% and 21% respectively [13,14]. However, given the potential for significant morbidity and mortality associated with delayed or incorrect diagnosis of *P. falciparum*, improvement is needed.

In this study, the majority of laboratories surveyed reported using both thick and thin smears; very few respondents reported the use of thin smears alone, an inferior technique. It is important that both thick and thin smears be evaluated in the examination of blood specimens for malaria. Thick smears are useful for detecting parasites, while thin smears are useful for species identification and quantification of parasites.

A few respondents reported that diagnostic testing was available only during the eight- to twelve-hour work day with no access to on-call personnel during the off-hours. Malaria is a potentially life threatening disease: therefore, it is recommended that all blood films be read immediately. CLSI recommends that 'any laboratory providing the expertise to identify malarial parasites should do so on a 24-hour basis, seven days a week' [11]. Therefore qualified personnel who can perform this testing should be on-call during off-hours.

Very few respondents reported availability of serologic testing in their laboratories; several other respondents reported its availability as a send-out test. Serologic testing is used only for retrospective diagnosis of past malaria infection [15] and is not recommended for diagnosis of acute infection. Anti-malarial antibodies are produced one to two weeks after initial infection and persist for three to six months after parasite clearance. Malaria RDTs are being used more frequently over the last several years, primarily in malaria-endemic areas[16]. Microscopy remains the gold standard, and in the U.S. is the primary and in some cases the only diagnostic tool used; as evidenced by the small number of respondents who reported having access to RDTs in their laboratories. RDTs can be beneficial in laboratories lacking personnel proficient in the microscopic diagnosis of malaria, as they allow for a rapid diagnosis of malaria, which can then be confirmed by microscopy or PCR. RDTs utilize immunochromatographic methods to detect malarial antigens present in peripheral blood to provide a quick result as to whether or not a patient is infected with malaria. Certain RDTs are able to give limited information about species (i.e. to indicate that at least *P. falciparum *is present) but none are able to provide quantification of parasitaemia. There are many brands and types of RDTs worldwide, but only one is FDA-approved for diagnostic use in the U.S.: BinaxNOW Malaria^® ^(Inverness Medical, Princeton, NJ). This test targets both the histidine-rich protein II (HRPII) antigen specific to *P. falciparum *and a malarial aldolase common to all human malaria species; it is therefore able to provide a diagnosis of malaria as well as determine specifically if *P. falciparum *is present[17]. However, all results must be confirmed by microscopy under the product's current FDA approval.

The detection of parasites in non-immune individuals who may be symptomatic at very low parasite densities is an additional barrier; unless enough fields are counted, these specimens may be mistakenly read as negative. In such cases it is imperative that repeat blood smears be performed. Standard guidelines recommend that a total of three specimens be obtained before declaring a patient does not have malaria. It is important to note that ensuring that an adequate number of specimens are sent is the responsibility of the physician; however, the laboratory may assist the physician with a reminder that three specimens must be sent to guarantee an accurate result.

Any laboratory with the capacity to perform a complete blood count with manual differential has the tools necessary to make a diagnosis of malaria and to report the percent parasitaemia (Additional file 2). All haematology technicians should have the basic training to identify a parasite on peripheral blood smear. Parasite speciation requires additional training and may therefore require sending the specimen to a reference laboratory. CDC provides diagnostic assistance, most rapidly (within 12-24 hours) through telediagnosis, where digital photographs and basic epidemiological data are sent to on-call personnel (See Additional file 2 for contact information*). Additionally, online diagnostic instructions are available at CDC's parasitology diagnostic website, DPDx [18] (See references for website address). For laboratories without on-site malaria diagnostic testing, consideration should be given to implementing RDTs, with confirmation via off-site testing.

Accurate identification of the infecting species has significant implications for treatment, as patients with *P. vivax *and *P. ovale *infections require therapy with primaquine to prevent relapses from latent hypnozoites in the liver. Accurate species identification is also important for detecting changes in the epidemiology of malaria among travellers. Laboratories should ensure that a definitive species-level diagnosis is made; PCR is a useful tool for definitively determining the *Plasmodium *species present. In addition, use of PCR should minimize the number of false negative and false positive results. PCR is more sensitive than microscopy for detecting mixed infections [19,20], as these are easily missed on a blood smear if morphologic features of both species are not visualized.

Although a high percentage of respondents reported compliance with each individual CLSI guideline, only very few representatives surveyed reported compliance with all CLSI guidelines. The purpose of these guidelines is to standardize practices across laboratories, as well as to recommend practices which will ultimately lead to the best outcomes through rapid identification and reporting of positive malaria smears. While species identification is critical to ensure that appropriate definitive treatment is given, providing a rapid presumptive diagnosis of malaria is most important to ensure prompt treatment is given. Therefore, while ideally all CLSI guidelines should be adhered to, those aimed at improving the rapidity and accuracy of initial reporting, such as counting an adequate number of fields and an adequate number of smears, are the most important.

**Figure 6:**
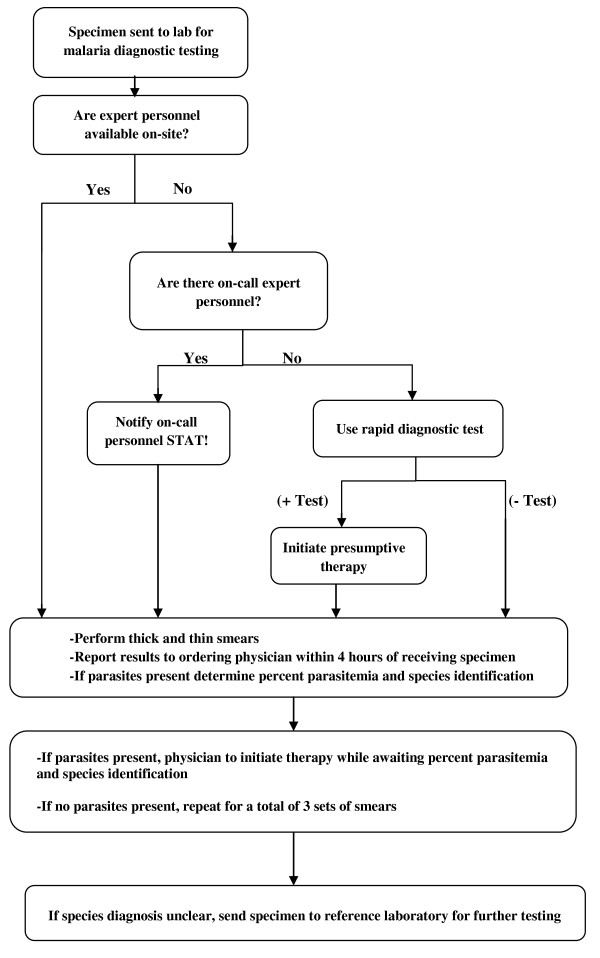
**Recommended Malaria Diagnostic Testing Algorithm**.

Figure 6 outlines a useful algorithm for laboratories receiving specimens for malaria diagnostic testing. A preliminary report of positive or negative should be available within 4 hours; percent parasitaemia should be reported within 6 hours and species identification within 24 hours. This may be more difficult in laboratories where tests are sent out; laboratory supervisors should facilitate rapid retrieval of these results to ensure physicians have the information necessary to optimally manage the patient. Otherwise, they should consider making RDTs available to facilitate the initial diagnosis. If this is not possible, specimens should be referred to a laboratory where malaria diagnostic testing can be performed in a timely fashion. Patient care is a collaborative effort which includes many members of the medical team. In addition to the vital role of the laboratory staff, it is paramount for physicians to obtain a complete history, including travel history, and keep malaria high on the differential diagnosis of patients presenting with fever. As physicians it is our responsibility to ascertain the availability of diagnostic tests in our institution's laboratories and, if they are lacking, advocate for more timely access to better malaria testing.

## Limitations

This survey was sent out to two listservs maintained by the American Society for Microbiology. As there is overlap in the membership of these lists and not all people on the list were eligible for participation (e.g. international members), it is impossible to calculate the true response rate, and our results may not be reflective of practices at all laboratories across the U.S. As with any survey, there is the potential for respondent bias. Although the responses received were from multiple different types of laboratories in a wide geographic distribution, it is possible that laboratories with more sophisticated testing for malaria or those who receive more samples for malaria may have been more likely to respond. Therefore, this data may represent the best case scenario rather than what occurs at the majority of laboratories.

The names of laboratories and affiliated institutions were not obtained; this was intentionally done in order to maintain the confidentiality of the respondents. However, this poses a limitation, as more than one member of a laboratory may have submitted answers to the survey. It is for this reason that the results are reported based on the number of respondents rather than the number of laboratories.

## Conclusions

The results of this survey attest to the availability of services for malaria diagnostic testing in U.S. laboratories, showing that the delays and accuracy of diagnosis may be due to lack of practice and proficiency as well as the difficulty in complying with the rigorous guidelines set by the CLSI. Even in laboratories with large volumes of samples processed for malaria parasite detection, each individual staff member likely sees very few cases per year; consequently, it is imperative that all laboratories, especially those that infrequently process samples for malaria seek opportunities for practice and proficiency training annually (Additional file 3).

## List of abbreviations used (in alphabetical order)

CDC: Centers for Disease Control and Prevention; CLSI: Clinical and Laboratory Standards Institute; DPDx: Division of Parasitic Disease Diagnosis; ELISA: Enzyme-linked immunosorbent assay; FDA: Food and Drug Administration; HPF: high power fields; HRP II: histidine-rich protein-2; IFA- indirect fluorescent antibody; PCR: polymerase chain reaction; RBCs: red blood cells; RDT: rapid diagnostic test.

## Competing interests

The authors declare that they have no competing interests.

## Authors' contributions

FA produced and disseminated the surveys, collected and analyzed the data, and drafted the manuscript. PA participated in the design of the survey and participated in drafting the manuscript. JG conceived of the study, participated in the design of the survey, and participated in drafting the manuscript. All authors read and approved the final manuscript.

## Supplementary Material

Additional file 1 **Malaria Diagnostic Survey Questionnaire**. Click here for file

Additional file 2 **Microscopic Procedures for Diagnosing Malaria**. Click here for file

Additional file 3 **Select Opportunities for Practice and Additional Training for Laboratory Diagnosis of Blood and Tissue Parasites**. Click here for file
