# Supplementary material for: State of malaria diagnostic testing at clinical laboratories in the United States, 2010: a nationwide survey
Source: Malar J. 2011 Nov 10;10:340. doi: 10.1186/1475-2875-10-340 (PMC3225402; doi:10.1186/1475-2875-10-340)
Supplement: Additional file 1 — Malaria Diagnostic Survey Questionnaire. [file 1475-2875-10-340-S1.DOC]

**MALARIA DIAGNOSTIC SURVEY QUESTIONNAIRE**

1. Which of these is the best description of your job title:
   1. Microbiologist
   2. Pathologist
   3. Clinical laboratory scientist (medical technologist)
   4. Other (please specify)
2. In which state is your laboratory located? (all US states and nine dependent territories listed as choices)
3. Which of these is the best description of your laboratory?
   1. University hospital
   2. Community hospital
   3. VA hospital
   4. Urgent care clinic
   5. Primary care center
   6. Commercial referral laboratory
   7. Other (please specify)
4. Do you offer diagnostic testing for malaria on-site?
   1. Yes, we offer testing in our laboratory
   2. No, tests are available in our laboratory, but we do send specimens to another laboratory for testing
5. How many specimens were sent to your laboratory for diagnostic testing in the last 12 months?
   1. 0
   2. 1-10
   3. 11-20
   4. 21-50
   5. 51-100
   6. >100
6. How many confirmed cases of malaria were diagnosed in the last 12 months at your hospital? (ie: malaria parasites detected by smear microscopy or PCR)
   1. 0
   2. 1-5
   3. 6-10
   4. 11-15
   5. >15

**Questions for laboratories with on-site testing (if answered ‘a’ to question 4):**

1. Which of the following tests do you offer either on site or as a send out lab? (Mark all that apply)

On-site Send out

Light microscopy- both thick and thin smears □ □

Antigen detection, also known as rapid diagnostic testing □ □

Serology by ELISA □ □

Serology by IFA □ □

PCR □ □

Other (please specify)

1. If microscopy is performed, what staining technique is used? (Mark all that apply)

- Giemsa stain
- Wright stain
- Wright-Giemsa stain
- Microscopy is performed as a send out only (skip to question #21)
- Other (please specify)

1. In your laboratory, how long does it typically take from receipt of specimen until a result (ie: parasite present or absent) is reported?
   1. <1 hour
   2. 1-4 hours
   3. 4-12 hours
   4. 12-24 hours
   5. 24-36 hours
   6. >36 hours
2. In your laboratory, how many slides from each sample are reviewed before the specimen is considered negative?
   1. 1
   2. 2
   3. 3 or more
   4. Don’t know
3. In your laboratory, how many high power fields (100x) in total are reviewed prior to determining a negative test?
   1. <100 HPF
   2. 101-149 HPF
   3. 150-299 HPF
   4. ≥ 300 HPF
   5. Don’t know
4. Who reviews the slides prior to reporting a NEGATIVE result?
   1. One laboratory technician reviews the smear prior to reporting a negative test
   2. Two laboratory technicians review the smear prior to reporting a negative test
   3. One laboratory technician + laboratory director review the smear prior to reporting a negative test
   4. One laboratory technician + pathologist review the smear prior to reporting a negative test
   5. Other (please specify)
5. Who reviews the slides prior to reporting a POSITIVE result?
   1. Two laboratory technicians review the smear prior to reporting a diagnosis of malaria
   2. One laboratory technician + laboratory director review the smear prior to reporting a diagnosis of malaria
   3. One laboratory technician + pathologist review the smear prior to reporting a diagnosis of malaria
   4. Comparison is made to a quality control smear only
   5. Other (please specify)
6. In your laboratory, how long does it typically take from receipt of specimen until PERCENT PARASITAEMIA is reported?
   1. < 1 hour
   2. 1-6 hours
   3. 6-12 hours
   4. 12-24 hours
   5. 24-36 hours
   6. >36 hours
   7. N/A, we report only whether the smear is positive or negative

If answer ‘g’ then skip to question #20

1. How many total red blood cells are counted to determine percent parasitaemia?
   1. <500 RBCs
   2. 500-1000 RBCs
   3. 1001-2000 RBCs
   4. 2001-3000 RBCs
   5. 3001-5000 RBCs
   6. >5000 RBCs
   7. Don’t know
2. Which department performs malaria screening and determines percent parasitaemia?
   1. Microbiology
   2. Hematology
   3. Pathology
   4. Don’t know/Not applicable
   5. Other (please specify)
3. In your laboratory, how is the SPECIES determined?
   1. Determined on site at our laboratory only
   2. Determined on site at our laboratory and confirmed at an outside laboratory (eg: state health department)
   3. Determined at a send out laboratory
   4. Other (please specify)
4. Who determines the DEFINITIVE species identification?
   1. Microbiology director or supervisor
   2. Hematologist
   3. Pathologist
   4. State Health Department
   5. Don’t know
   6. Other (please specify)
5. How long does it typically take from receipt of specimen until SPECIES of parasite is reported?
   1. < 1 hour
   2. 1-6 hours
   3. 6-12 hours
   4. 12-24 hours
   5. 24-36 hours
   6. 36-48 hours
   7. 48-72 hours
   8. 3-5 days
   9. 5-7 days
   10. >7 days
   11. Don’t know
6. What is the availability of malaria diagnostic testing in your laboratory?
   1. Available during working hours (9 am- 5pm or equivalent 8 hour period) only
   2. Available during working hours (9 am- 5pm or equivalent 8 hour period) and after working hours by request only
   3. Available 24 hours, 7 days a week
   4. Other (please specify)

**Questions for laboratories which only send out testing (if answered ‘b’ to question 4):**

1. Which of the following tests are offered as send outs? (Mark all that apply)
   - Light microscopy- thick smear only
   - Light microscopy- thin smear only
   - Light microscopy- both thick and thin smears
   - Antigen detection, also known as rapid diagnostic testing
   - Serology by ELISA
   - Serology by IFA
   - PCR
   - Other (please specify)
2. If diagnostic tests are performed at an outside laboratory, how long does it typically take before the level of parasitaemia and speciation of parasite is reported back to your laboratory?
   1. Within 24 hours
   2. 24- 48 hours
   3. 48-72 hours
   4. 3- 5 days
   5. 5- 7 days
   6. > 7 days
