# Supplementary material for: State of malaria diagnostic testing at clinical laboratories in the United States, 2010: a nationwide survey
Source: Malar J. 2011 Nov 10;10:340. doi: 10.1186/1475-2875-10-340 (PMC3225402; doi:10.1186/1475-2875-10-340)

**ADDITIONAL FILE 2**

**Microscopic Procedures for Diagnosing Malaria**

To establish the diagnosis of malaria, a blood film must be prepared from fresh blood obtained by pricking a patient’s finger with a sterile, non-reusable lancet (see Additional File 2, Figure 1). Two types of blood films can be used: thin films (as used for hematology) and thick films. Thick and thin films can be made as separate or as combination slides (see Additional File 2, Figure 2). Thick blood films are more sensitive in detecting malaria parasites because the blood is concentrated, allowing a greater volume of blood to be examined. However, thick films are more difficult to read. Thin films allow for species and parasite density determinations.

The thin film should be air-dried, fixed with methanol, and allowed to dry before staining; the thick film also should be thoroughly dried but stained without fixation. For best staining results, blood films should be stained with a 2.5% Giemsa solution (pH of 7.2) for 45 minutes (alternate: 7.5% Giemsa for 15 minutes). A combined Wright-Giemsa stain can also detect malaria parasites but does not demonstrate Schüffner’s dots as reliably as Giemsa.

*Plasmodium* parasites are always intracellular, and they demonstrate, if stained correctly, blue cytoplasm with a red chromatin dot. Common errors in reading malaria films can be caused by platelets overlying a red blood cell, concern regarding missing a positive slide, and misreading of artifacts as parasites. In *P. falciparum* infections, the parasite density should be estimated by counting the percentage of red blood cells infected (not the number of parasites) under an oil immersion lens on a thin film (see Additional File 2, Figure 3).

Persons suspected of having malaria, but whose blood films do not indicate the presence of parasites, should have blood films repeated approximately every 12–24 hours for a total of 3 sets. If films remain negative, then the diagnosis of malaria is unlikely. A useful complement to microscopy can be found in polymerase chain reaction (e.g., for determining parasite species). Additional information regarding collection and preparation of blood films is available at CDC’s Internet site, DPDx — Laboratory Identification of Parasites of Public Health Concern (<http://www.dpd.cdc.gov/DPDx>).

*Digital photographs and basic epidemiological data can be sent to [dpdx@cdc.gov](mailto:dpdx@cdc.gov) for diagnostic assistance.

**Figure 1. Blood Collection Procedure for Thick and Thin Film Preparation.**

**Figure 2. Preparation of Thick and Thin Smears for Malaria Diagnosis.**

**Figure 3. Malaria Smear Demonstrating *P. falciparum* Infection with Multiply Infected Red Blood Cells.**  Percent parasitaemia is calculated by dividing the number of infected RBCs by total number counted (should be 500 – 2,000 RBCs) and then multiplying by 100. Note that those cells infected with more than one ring form, as demonstrated in this picture, are only counted once.

Percent parasitaemia= [(number of infected RBCs)/ (total RBCs counted)] * 100


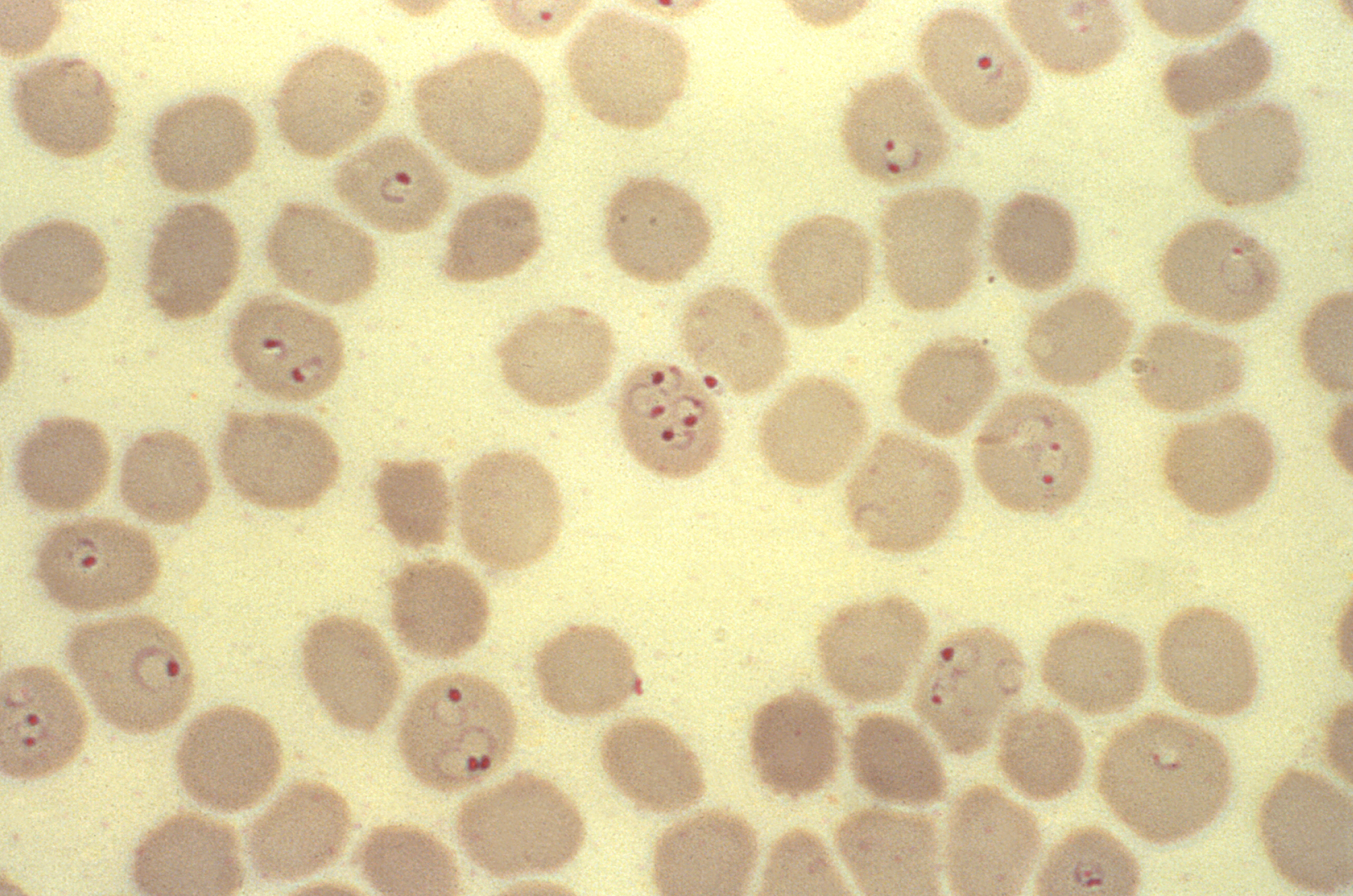

Supplement: Additional file 2 — Microscopic Procedures for Diagnosing Malaria. [file 1475-2875-10-340-S2.DOC]
