# Supplementary material for: State of malaria diagnostic testing at clinical laboratories in the United States, 2010: a nationwide survey
Source: Malar J. 2011 Nov 10;10:340. doi: 10.1186/1475-2875-10-340 (PMC3225402; doi:10.1186/1475-2875-10-340)
Supplement: Additional file 3 — Select Opportunities for Practice and Additional Training for Laboratory Diagnosis of Blood and Tissue Parasites. [file 1475-2875-10-340-S3.DOC]

**ADDITIONAL FILE 3**

**Select Opportunities for Practice and Additional Training for Laboratory Diagnosis of Blood and Tissue Parasites**

**On-line tutorial:**
Para-Site Online (includes quizzes):
[http://www.med-chem.com/Para/index.htm](https://owalegacy.emory.edu/owa/redir.aspx?C=1ef81cc2f3164c3298b4e2bc1713c4dd&URL=http%3A%2F%2Fwww.med-chem.com%2FPara%2Findex.htm)

**National meeting workshops:**
-American Society for Microbiology's workshop on "Laboratory Diagnosis of Blood and Tissue Parasites" ([http://gm.asm.org/index.php/scientific-program/workshops/workshop-descriptions](https://owalegacy.emory.edu/owa/redir.aspx?C=1ef81cc2f3164c3298b4e2bc1713c4dd&URL=http%3A%2F%2Fgm.asm.org%2Findex.php%2Fscientific-program%2Fworkshops%2Fworkshop-descriptions))

-Association of Public Health Laboratories' Hands-On Laboratory Workshops ([http://www.aphl.org/profdev/training/handson/pages/default.aspx](https://owalegacy.emory.edu/owa/redir.aspx?C=1ef81cc2f3164c3298b4e2bc1713c4dd&URL=http%3A%2F%2Fwww.aphl.org%2Fprofdev%2Ftraining%2Fhandson%2Fpages%2Fdefault.aspx))
 CDC Atlanta/NLTN Hands-On Laboratory Workshop

**Other resources:**
(retrieved from DPDx Training site- [http://www.dpd.cdc.gov/dpdx/HTML/Frames/OtherDocs/body_training.htm](https://owalegacy.emory.edu/owa/redir.aspx?C=1ef81cc2f3164c3298b4e2bc1713c4dd&URL=http%3A%2F%2Fwww.dpd.cdc.gov%2Fdpdx%2FHTML%2FFrames%2FOtherDocs%2Fbody_training.htm))
**Malaria Microscopy Reference Slides Available Through MR4:**
Microscopy reference slides sets on malaria are available through the Malaria Research and Reference Reagent Resource Center on a loan-only basis.  For more information on how to qualify to receive a loaned slide set, please see MR4's website [http://www.mr4.org/](https://owalegacy.emory.edu/owa/redir.aspx?C=1ef81cc2f3164c3298b4e2bc1713c4dd&URL=http%3A%2F%2Fwww.mr4.org%2F) under "Microscopy Slides."

**DPDx Monthly Case Studies and Archive:**
DPDx offers a monthly Parasitology Case Study Quiz by email.  The purpose of this service is to provide distance training on morphologic diagnostic parasitology to professionals engaged in identifying parasites associated with human disease.  On the third week of each month, two case studies are sent to subscribers via email as well as posted on the Monthly Case Studies page of the DPDx website.  Within a week, subscribers should send their answers to DPDx at dpdx@cdc.gov in order to have a valid participation in this CDC training activity.  The correct answers to the case studies are released the following week; they are sent by email as well as posted on the DPDx website.  Feel free to contact the DPDx Team (dpdx@cdc.gov) if you have any questions regarding the DPDx Monthly Case Studies.
